# Motor Improvement of Skilled Forelimb Use Induced by Treatment with Growth Hormone and Rehabilitation Is Dependent on the Onset of the Treatment after Cortical Ablation

**DOI:** 10.1155/2018/6125901

**Published:** 2018-03-20

**Authors:** Margarita Heredia, Jesús Palomero, Antonio de la Fuente, José María Criado, Javier Yajeya, Jesús Devesa, Pablo Devesa, José Luis Vicente-Villardón, Adelaida S. Riolobos

**Affiliations:** ^1^Department of Physiology and Pharmacology, Neuroscience Institute of Castilla y León (INCyL), University of Salamanca, Salamanca, Spain; ^2^Scientific Direction, Medical Centre Foltra, Teo, Spain; ^3^Research and Development, Medical Centre Foltra, Teo, Spain; ^4^Department of Statistics, University of Salamanca, Salamanca, Spain

## Abstract

We previously demonstrated that the administration of GH immediately after severe motor cortex injury, in rats, followed by rehabilitation, improved the functionality of the affected limb and reexpressed nestin in the contralateral motor cortex. Here, we analyze whether these GH effects depend on a time window after the injury and on the reexpression of nestin and actin. Injured animals were treated with GH (0.15 mg/kg/day) or vehicle, at days 7, 14, and 35 after cortical ablation. Rehabilitation was applied at short and long term (LTR) after the lesion and then sacrificed. Nestin and actin were analyzed by immunoblotting in the contralateral motor cortex. Giving GH at days 7 or 35 after the lesion, but not 14 days after it, led to a remarkable improvement in the functionality of the affected paw. Contralateral nestin and actin reexpression was clearly higher in GH-treated animals, probably because compensatory brain plasticity was established. GH and immediate rehabilitation are key for repairing brain injuries, with the exception of a critical time period: GH treatment starting 14 days after the lesion. Our data also indicate that there is not a clear plateau in the recovery from a brain injury in agreement with our data in human patients.

## 1. Introduction

Brain repair after an injury involves a number of complex processes. Abundant evidence indicates that growth hormone (GH) administration, added to rehabilitation, can significantly contribute to the recovery of an acquired brain injury, both in animal models [1–9] and in human patients [10–15], regardless of whether the patient is GH-deficient (GHD) or not [14–18]. However, it is not clear whether there is a period of time after a brain injury during which GH can exert its positive effects for brain repair. While it seems logical that early GH administration and rehabilitation after brain damage should provide faster and better recovery [14–16], recent data from our group demonstrate that brain repair in humans can be achieved by administering GH together with rehabilitation even months or years after the injury happened [10, 16–18]. These data challenge the classical concept that there is a “plateau” for brain recovery following an injury after which few more positive improvements could be obtained.

In a previous work, we demonstrated, in rats, that GH administration, but not vehicle, given immediately after a severe lesion of the frontal motor cortex led to a significant improvement of the motor impairment induced by frontal cortical ablation of the dominant hemisphere. Therefore, their performance in the paw-reaching-for-food task was soon similar to that in sham-operated controls [19]. However, in the same study, despite intense rehabilitation, no significant changes were observed in motor function of animals receiving vehicle or GH 6 days after induction of a severe injury in the frontal motor cortex. This is perhaps because in this case, rehabilitation commenced 13 days after the lesion was produced, that is, almost at the end of the critical period of time in which rehabilitative therapies may achieve maximal efficiency in rats (5 to 14 days after an injury) [20]. It has been shown that rehabilitative therapies initiated during these days enhance dendritic growth in the undamaged motor cortex [20], but the heightened sensitivity to rehabilitation commencing early after the lesion declines with time [20].

In our previous study, we detected reexpression of nestin in the contralateral motor cortex only in injured rats treated with GH. This reexpression of nestin was clearly higher in animals receiving the hormone immediately after the injury than in rats in which GH treatment commenced 6 days after the injury occurred [19]. Since the substantial magnitude of the lesion made it impossible for the injured area to regenerate, we believe that the reexpression of nestin in the contralateral motor cortex of animals treated with GH (not observed in animals treated with vehicle) would be the factor responsible for explaining the differences in the recovery observed between the different groups of animals. Moreover, we deduced that GH administration has to be followed by early rehabilitation in order to obtain significant motor improvements [19].

Nestin expression is commonly used as a marker of neural stem and progenitor cells. However, nestin is expressed in many other nonneural progenitor cell types, such as developing muscle, endothelial cells, and reactive astrocytes, especially in the injured brain [21–26]. Therefore, it is likely that nestin reexpression in GH-treated rats played a significant role in mechanisms of brain plasticity, leading to the quick improvement observed in rats treated with GH and rehabilitation immediately after the lesion occurred. Meanwhile, the fact that the expression of this neural marker was lower in the contralateral cortex of GH-treated animals in which rehabilitation after GH administration commenced at the end of the critical period for maximal efficiency of rehabilitative therapy suggests that GH induces the reexpression of nestin. However, it also suggests that a number of factors (which are elevated towards the end of the critical period) can prevent GH expression from reaching the magnitude required to achieve significant brain plasticity after a brain injury.

It is well known that any damage to the adult brain generates an adaptative plasticity which depends on neurogenesis [27] and new axonal connections [28, 29]. It has been seen, in rodents, that stroke induces proliferation of newly born neurons in the subventricular zone (SVZ), migration of these immature neurons away from the SVZ, and localization within peri-infarct tissues. Immature neurons migrate after a stroke in close association with blood vessels and astrocytic processes [29]. This poststroke migration is very similar to the normal neuroblast migration in the rostral migratory stream. Immature neurons localize in the peri-infarct cortex in a neurovascular niche where neurogenesis is causally linked to angiogenesis through a number of vascular and neuronal growth factors including erythropoietin (EPO) [29]. Interestingly, most of these factors, if not all of them, are induced by GH [16]. However, despite the fact that for many years the expression of GH and its receptor has been detected in the brain [30]—where its neuroprotective role is well known [3]—the mechanisms by which the hormone acts and the time period during which these mechanisms may play a positive role still remain to be elucidated.

In this study, we analyzed whether the administration of GH immediately followed by rehabilitation in different periods of time after induction of severe ablation of the frontal motor cortex was able to induce significant motor improvements. That is, we studied whether there is a critical window of time for GH action, together with rehabilitation, in brain recovery after an injury in rats. Our results indicate that the administration of GH plus rehabilitation at days 7 or 35 after the lesion, but not rehabilitation alone, significantly improve the lost motor function. However, this did not occur when the GH treatment was given 14 days after the cortical ablation was produced, in spite of the fact that the rehabilitation commenced on that same day in the other GH-treated groups. In addition, our data also suggest that rehabilitation has to be performed immediately after GH therapy begins in order to achieve significant motor improvements. Moreover, in the motor cortex of the undamaged hemisphere, we observed an important reexpression of nestin and actin in GH-treated rats, but not in vehicle-treated or control animals, which probably indicates the development of compensatory mechanisms to achieve a functional recovery.

## 2. Materials and Methods

Thirty-five adult male Wistar rats (Charles River Laboratories, Spain), with body weight 200–220 g at the beginning of the experiments, were used for the behavioral and nestin/actin Western blot experiments. Animals were housed under controlled conditions of temperature (18–20°C) and natural light/dark cycles, at least 4 days prior to the experiments. They were fed with a normal chow diet and water *ad libitum*, except when the paw-reaching-for-food task was carried out. At this time, animals were maintained at 86–88% of their initial *ad libitum* weight. All experiments and procedures involved in this study were approved by the University of Salamanca Ethics Committee and were conducted in accordance with the animal care European guidelines (2010/63/EU) and Spanish regulations (Real Decreto 53/2013). In addition, every effort was made to minimize the suffering and number of animals used.

### 2.1. Experimental Design for Behavioral Test

The experimental design consisted of the following phases:

1. *Paw-reaching-for-food task. Presurgical phase*. Animals were trained in the paw-reaching test, and the preferred paw forelimb was recorded
2. *Ablation of frontal motor cortex. Evaluation of the effectiveness of the lesion.* Anaesthetized animals were lesioned by aspiration in the motor cortex contralateral to the preferred paw or sham-operated. The effectiveness of the lesion was then verified at day 7 postablation
3. *Treatment with GH or vehicle and rehabilitative therapies* (forced use of the affected paw) *at different times after cortical ablation. Evaluation of the paw-reaching-for-food responses*
4. Analysis of the nestin and actin expression in the motor cortex of the undamaged hemisphere by Western blot

**Figure 1:**
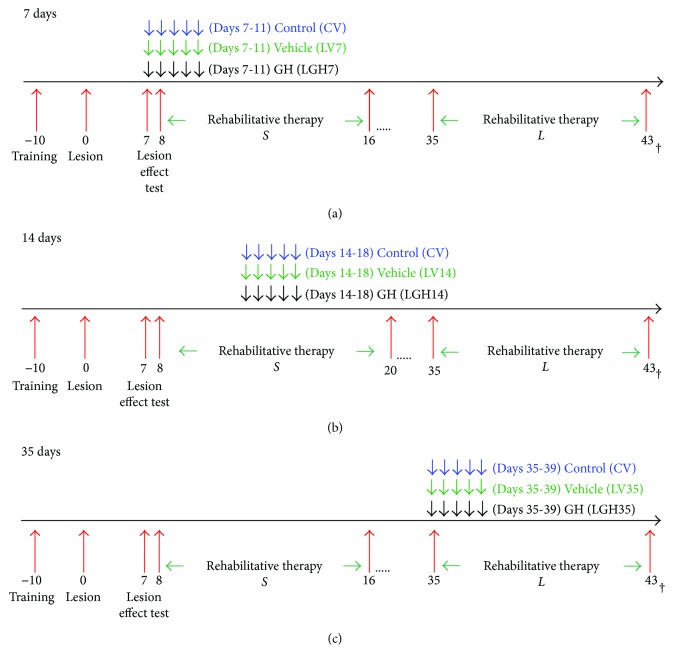
Schematic diagram of the experimental design. In (a), (b), and (c), −10 to 0 represent the days of training of the animals for paw-reaching-for-food task and to record the preferred forelimb (preoperative stage). In day 0, the motor cortex contraleral to the preferred limb was lesioned by aspiration. Arrows indicate the days during which GH or vehicle was given to each group of animals. Two groups of lesion animals (not GH treated) were given vehicle: in one group, it was administered on days 7 and 35 postablation (altogether called LV7/LV35 group) and in another group on day 14 (LV14 group) postablation. *S* indicates the days during which animals received short-term rehabilitative therapy, while *L* indicates the days during which animals received long-term rehabilitative therapy. 17 to 35 in (a), 21 to 35 in (b), and 17 to 35 in (c) correspond to resting period days (animals were kept in their cages without receiving any treatment). 43 correspond to the last day of long-term rehabilitative therapy.

All surgical procedures and sacrifices were carried out under deep anesthesia with Equithesin (20 mg/kg, intraperitoneally). These study phases are shown schematically in Figure 1.

#### 2.1.1. Paw-Reaching-for-Food Task. Presurgical Phase

Seven days after the arrival of animals, they were trained for the paw-reaching-for-food task: a specific motor test in which animals are conditioned to perform high-precision motor movements of extension and flexion of the forelimb fingers in order to obtain food. This test for fine motor skills has been widely used in previous studies from our group [19, 31–33]. Before carrying out the test, animals were housed individually and food was restricted until their body weight decreased to 86–88% of their previous *ad libitum* body weight. The design of the test cage has been described in previous studies of our laboratory [19, 31–33].

**Figure 2:**
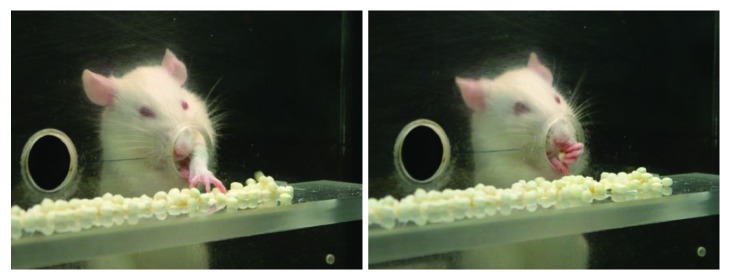
Consecutive photographs illustrating a rat in the test cage showing successful responses in the paw-reaching test during training in the presurgical phase. The design of the test cage prevented use of the tongue to retrieve food pellets or to rake the pellets.

In the paw-reaching-for-food test, rats were required to extend a forelimb through the hole, grasp and retrieve a pellet from the groove, take it to their mouth, and eat it (Figure 2). Each time an animal succeeded in eating a pellet without dropping it was counted as a successful response, while any failure in the sequence for obtaining food was counted as an unsuccessful response.

In this presurgical phase, each experimental animal was placed in the test cage in individual sessions lasting 3 minutes for 10–12 sessions, in order to quantify the number of successful and unsuccessful responses with both paws. During this phase, the preferred paw (right or left) of each animal was established. The total number of responses (successful and unsuccessful with both paws) and the percentage of successful responses with the preferred paw with regard to the total number of responses were recorded.

This motor test was used in the presurgical phase to train the animals and to establish which was the preferred paw, after inducing the lesion (to test its efficiency) and also during the rehabilitative therapies (Figure 1).

#### 2.1.2. Ablation of Frontal Motor Cortex

**Figure 3:**
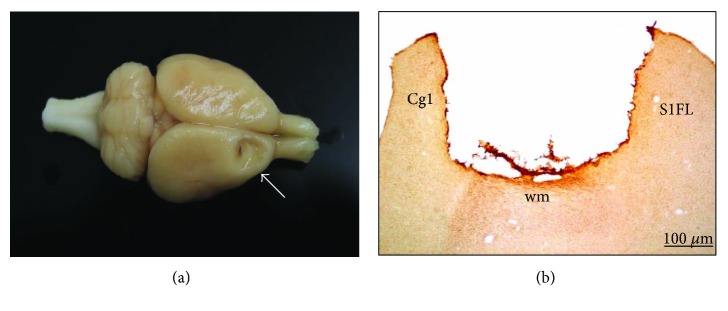
(a) Photograph of an example of a rat brain with motor cortex ablation (white arrow). (b) Photomicrograph of a brain coronal section showing the motor cortex lesion. Cg1: cingulated cortex, area 1; S1FL: primary somatosensory cortex, forelimb; wm: white matter. Scale bar = 100 *μ*m.

Each rat was placed in a stereotaxic apparatus and the skull exposed at the level of bregma. Animals were divided at random into two groups. One group (*n* = 30) was subjected to unilateral frontal cortex lesion. The other group (*n* = 5) was sham operated. Cortical ablation was performed, as in our previous work [19], at the coordinates indicated by Neafsey et al. [34], to remove the forelimb area of the motor cortex. A section of the skull was removed unilaterally 1 to 4 mm anterior to bregma and 1 to 3.5 mm lateral to the midline. The corpus callosum established the ventral limits of the lesion.  Figure 3 shows an example of a motor cortex ablation.

Lesions were caused by aspiration in the hemisphere contralateral to the preferred paw determined in the presurgical phase. Under visual guidance, and using an operating microscope, meninges were removed and a glass pipette connected to an aspiration pump was gently introduced into the cortex to remove the tissue. Care was taken to spare the white matter underlying the cortex. Cortical ablation was severe and homogenous in size and localization and is similar to that made in previous studies from our group [19, 31–33]. Lesions were restricted to the primary and secondary motor cortex areas (M1 and M2), although in some cases, the cingulate cortex, area 1, (Cg1) was slightly affected (Figure 3(b)). After surgery, the skin was sutured.

Control animals were subjected to the same surgical process in the contralateral motor cortex to the preferred forelimb, except for the lesion-inducing procedure itself (sham-operated group).

After surgery, the paw-reaching-for-food task established whether the lesion had been effective: either animals began to use their nonpreferred paw to reach for food, or the percentage of successful responses with the preferred paw was significantly decreased with regard to previous values in the presurgical phase.

#### 2.1.3. Treatment with GH or Vehicle and Rehabilitative Therapies (Forced Use of the Affected Paw) at Different Times after Cortical Ablation. Evaluation of the Paw-Reaching-for-Food Responses

In three groups of injured animals, rhGH (Saizen, Merck; 0.15 mg/kg/day, subcutaneously) was administered for 5 consecutive days commencing on day 7, day 14, or day 35 after cortical ablation (Figure 1) (LGH7 group, *n* = 7; LGH14 group, *n* = 6; and LGH35 group, *n* = 7, resp.).

In order to minimize the number of animals used, according to the European guidelines, two other groups of lesion animals (*n* = 5 per group, not GH treated) were given vehicle (phosphate buffer saline 0.1 M, pH 7.4, PBS). In one of these groups, it was administered on day 7 and day 35 (altogether called LV7/LV35 group), and in the other group, on day 14 (LV14 group) postablation. Vehicle was administered for 5 days starting at each time point. For control purposes, the sham-operated animals received vehicle for 5 days starting on days 7, 14, and 35 after sham operation (CV group, *n* = 5). In this study, we did not include a group of sham-operated animals treated with GH, because this had been done in our previous work without finding differences between sham-operated animals treated with GH or vehicle [19]. GH or vehicle treatments were administered conjointly with the rehabilitation therapy (commencing on days 8 or 35; Figure 1).

Rehabilitative therapy consisted in inducing the forced use of the forelimb affected by the frontal motor cortex lesion (preferred paw), by attaching a removable plaster bracelet to the forelimb of the nonpreferred paw (undamaged paw, ipsilateral to the lesion), which prevented reaching food but not other movements. Therefore, animals could reach food only with the impaired paw (preferred paw, contralateral to the lesion) [19]. The animals wore the bracelet only during the test and not continuously. Rehabilitative therapy was carried out for 9 consecutive days in daily sessions of 3 minutes. Rehabilitative therapy was applied to all experimental groups (including sham-operated controls), at two different time points after cortical ablation: short-term rehabilitation and long-term rehabilitation.

Short-term rehabilitation commenced on day 8 after the lesion. The first rehabilitation period lasted 9 days in the LGH7, LGH35, and LV7/LV35 group and 13 days in the LGH14, LV14, and CV groups (*S* in Figure 1). Long-term rehabilitation commenced on day 35 after cortical ablation. This second rehabilitative therapy lasted 9 days (*L* in Figure 1).

During the period of time comprised between short- and long-term rehabilitation, the animals were kept in their cages and food and water were freely available (Figure 1).

#### 2.1.4. Analysis of the Nestin and Actin Expression in the Motor Cortex of the Undamaged Hemisphere by Western Blot

**Figure 4:**
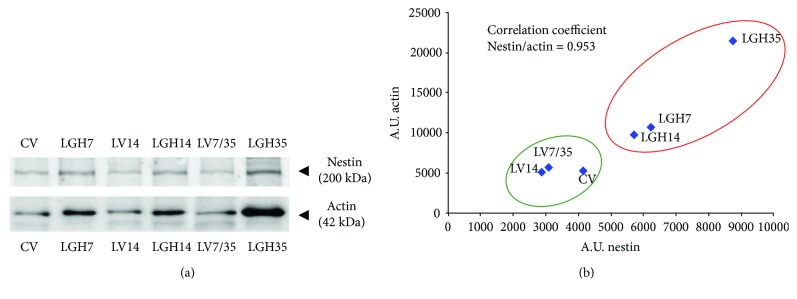
(a) Immunoblot images of nestin and actin expression in the motor cortex of the undamaged hemisphere, at 51 days postablation. Results show nestin and actin expression in homogenates of motor cortex samples from animals that were under different GH treatments (LGH7, LGH14, and LGH35) or controls (CV, LV14, and LV7/35). In each immunoblot band, either nestin or actin is identified with its corresponding GH-treated animal or control, and it comes from one animal in each case. Nestin and actin molecular weight, kilodaltons (kDa), is indicated. (b) Densitometric analysis of immunoblot images of nestin and actin displayed in (a). Arbitrary units (A.U.) of densitometry. Blue points represent the values (densitometric arbitrary units from immunoblot bands) of nestin and actin expression in each motor cortex sample that comes from one animal. Red underlined ellipse identifies samples from animals treated with GH, and green underlined ellipse identifies samples from control animals.

Animals treated with GH or vehicle at 7, 14, or 35 days postablation, and sham-operated control animals were deeply anesthetized with Equithesin and sacrificed by decapitation. Brains were immediately removed, and a sample of the motor cortex from the undamaged hemisphere, approximately 3 mm^3^, was dissected out, immediately snap-frozen in liquid nitrogen and then stored at −80°C. Motor cortex samples were individually homogenized in homogenization buffer 10 mM TRIS–HCl pH 7.4, 100 mM NaCl, 1 mM EDTA, 1 mM EGTA, 1% (*v*/*v*) Triton X-100, 10% (*v*/*v*) glycerol, 0.1% (*w*/*v*) dodecyl sodium sulfate, 0.5% (*w*/*v*) deoxycholate, and protease inhibitor cocktail (Sigma-Aldrich). Sample homogenates were centrifuged, and supernatants were collected in order to obtain the protein samples to be resolved by electrophoresis. Total protein content was determined using the bicinchoninic acid kit for protein determination (Sigma-Aldrich), and then samples were stored at −80°C until electrophoresis was performed. For electrophoresis, 30 *μ*g of each protein sample was mixed with an equal volume of loading buffer (2X Laemmli Buffer). Then this mixture was incubated at 95°C for 10 min to obtain the loading samples, which were loaded in a SDS-PAGE gel (4–10% polyacrylamide) together with molecular weight protein markers (Bio-Rad). Then electrophoresis was performed (200 V for 45 min), and resolved proteins were transferred to a nitrocellulose membrane by applying 100 V, 350 mA for 60 min. No specific binding sites in the membranes were blocked by a 1-hour incubation with 5% (*w*/*v*) powdered nonfat milk. Membranes were incubated overnight at 4°C with the primary antibody diluted (1 : 1000) in TBS-T with 1% bovine serum albumin (BSA). The primary antibodies were anti-nestin (clone Rat 401, Millipore, MAB353) and anti-actin (polyclonal rabbit antibody, Sigma, A2103). Both anti-nestin antibody and anti-actin antibody were probed on independent membranes from independent gels. Membranes were incubated with the secondary antibodies, peroxidase-conjugated goat anti-mouse (Jackson ImmunoResearch, 115–035-174) (dilution 1 : 10,000), or donkey anti-rabbit (Amersham, NA934VS) (dilution1 : 10,000). Membranes were incubated with a chemiluminiscence reagent (ClarityTM Western ECL Blotting Substrate, Bio-Rad), and proteins were visualized by chemiluminiscence using a digital recording CCD imaging system (MicroChemi, Bio-Imaging Systems).  Figure 4(a) shows digital images of nestin and actin. In addition, image densitometric analysis (using software ImageJ 1.46r, National Institute of Health, USA) was carried out to quantify the expression of nestin and actin in each motor cortex sample and presented in Figure 4(b), where each point identifies the expression of nestin and actin in motor cortex from an animal treated with GH, vehicle, or sham-operated control. In addition, the correlation coefficient (*R*) between nestin and actin was calculated. This coefficient indicates the direct relationship of the expression of both proteins in the different groups of animals.

### 2.2. Data Analysis

Statistical analysis was performed by using the Statview and SPSS programs. Each group of animals treated with GH at each time point was compared to the corresponding vehicle-treated group and sham-operated control group (CV). The percentage of successful responses with the preferred paw and the total number of responses (successful + unsuccessful with both paws) were compared. Fine motor skills results were analyzed by repeated ANOVA measures (group and session). Together with the *P* values, the eta squared (*η*^2^) measure for the effect size was reported. Eta squared values higher than 0.26 are considered as strong effect size in the literature. When global ANOVA showed a significant difference among groups (*P* ≤ 0.05), partial ANOVA comparing mean values and standard deviation of the different groups in each session was performed. To compare individual means, the Bonferroni post hoc test was used; *P* < 0.016 was the value considered as the limit for significance among groups. The relationship between the amount of nestin and actin detected in electrophoresed samples was calculated, after performing a densitometric analysis. The calculation of correlation coefficient from densitometric data was performed by Microsoft® Excel® software. The value of this coefficient indicates the direct relationship between the expression of nestin and actin in animals treated with GH or vehicle or sham-operated control.

## 3. Results

**Figure 5:**
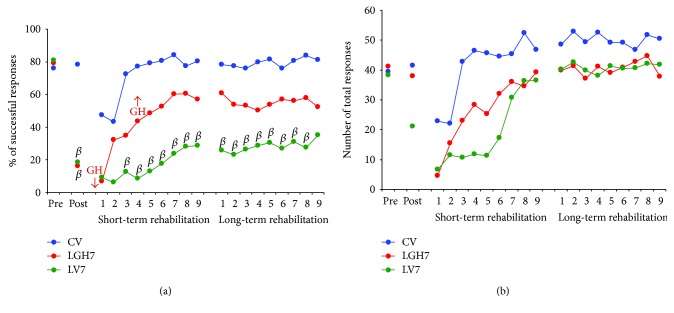
Animals treated with GH or vehicle at 7 days after cortical ablation. Behavioral results obtained in the paw-reaching-for-food task with the preferred paw (impaired paw) at the presurgical phase (PRE), postablation (POST), and rehabilitative therapies. (a) Mean percentage of successful responses (successful responses/total number of responses). (b) Mean of the total number of responses (successful and unsuccessful with both paws). The rehabilitative therapies consisted in the forced use of the impaired paw, in daily sessions for 3 min during 9 consecutive days (indicated in *x*-axis). No differences existed between LGH7 animals and sham-operated controls (CV), while a clear lack of successful responses was found in lesion animals treated with vehicle (LV7) as compared to both GH-treated animals and controls. Significant levels are obtained after comparison with sham-operated controls (CV). *β* = *P* < 0.01 (Bonferroni's test). GH arrows indicate when GH treatment commenced and finished in LGH7 animals.

**Figure 6:**
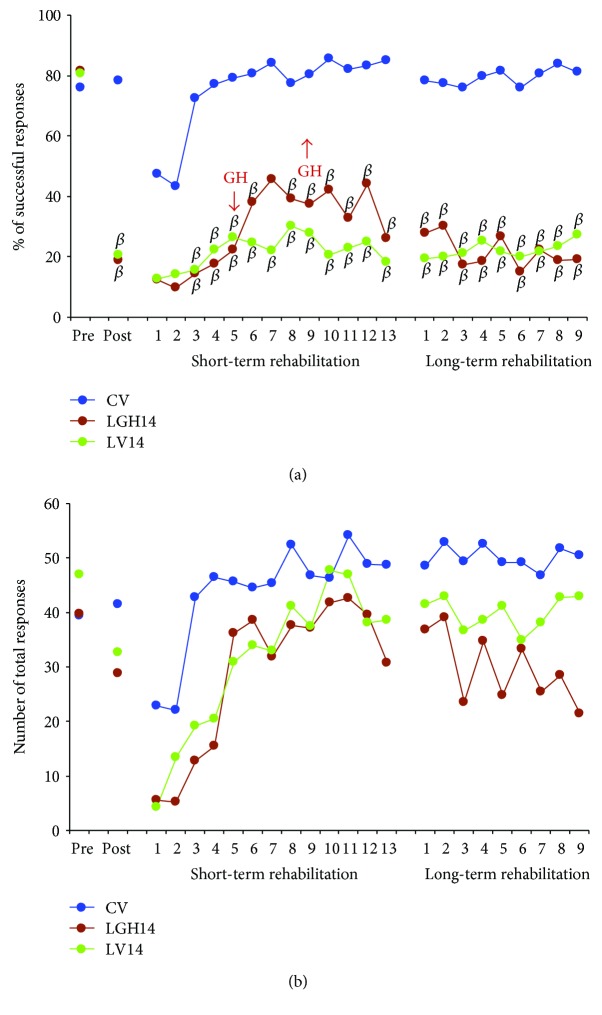
Animals treated with GH or vehicle at 14 days after cortical ablation. Behavioral results obtained in the paw-reaching-for-food task with the preferred paw (impaired paw) at the presurgical phase (PRE), postablation (POST), and rehabilitative therapies. (a) Mean percentage of successful responses (successful responses/total number of responses). (b) Mean of the total number of responses (successful and unsuccessful with both paws). The rehabilitative therapies consisted in the forced use of the impaired paw, in daily sessions for 3 min during 13 (short-term rehabilitation) and 9 (long-term rehabilitation) consecutive days (showed in *x*-axis). Significant levels (the Bonferroni post hoc test) indicated that both vehicle-treated animals (LV14) and GH-treated rats did not improve their percentage of successful responses in comparison to results obtained in the sham-operated control group (CV). *β* = *P* < 0.01; GH arrows indicate when GH treatment commenced and finished in LGH14 animals.

**Figure 7:**
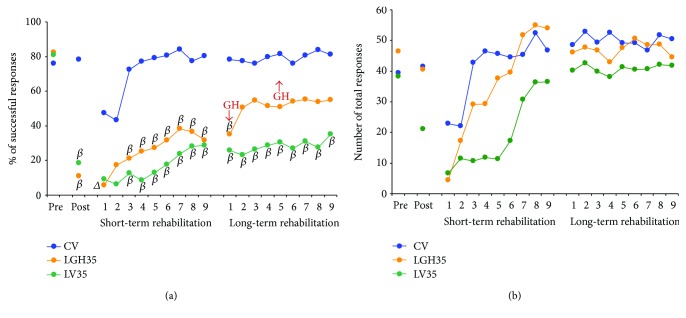
Animals treated with GH or vehicle at 35 days after cortical ablation. Behavioral results obtained in the paw-reaching-for-food task with the preferred paw (impaired paw) at the presurgical phase (PRE), postablation (POST), and rehabilitative therapies. (a) Mean percentage of successful responses (successful responses/total number of responses). (b) Mean of the total number of responses (successful and unsuccessful with both paws). The rehabilitative therapies consisted in the forced use of the impaired paw, in daily sessions for 3 min during 9 consecutive days indicated in (*x*-axis). GH-treated animals (LGH35) improved their percentage of successful responses after the second session, reaching a value no longer different to that of the sham-operated control group (CV), while vehicle-treated animals (LV35) did not change their low percentage of successful results. Significant levels are obtained after comparison with sham-operated controls (CV). *β* = *P* < 0.01; *Δ* = *P* < 0.05 (Bonferroni's test). GH arrows indicate when GH treatment commenced and finished in LGH35 animals.

Figures 5, 6, and 7 show the results from the paw-reaching-for-food task at the three different time points of GH or vehicle treatment (7, 14, and 35 days postablation, resp.), during the different phases of the experiment: (a) mean percentages of successful responses obtained with the preferred paw with regard to the total number of responses; (b) average of the total number of responses (successful plus unsuccessful with both paws).

### 3.1. Presurgical Phase

In this phase, the ability to take food pellets from the groove and eat them was similar in all the rats. All rats displayed a stable strategy using a single forelimb to reach the pellets; thereby, a spontaneous limb preference was established during the training period. In all animals, limb preference was established at the fourth session, and the paw used was then considered the preferred paw. When the percentage of attempts using the right or left paw was between 85 and 100%, the rat was classified as right- or left-handed. It was considered that animals were well trained when the percentage of successful responses was ≥60% during two consecutive sessions. To distribute animals into the different experimental groups, the mean of results obtained in the two last sessions of this phase was taken. Therefore, both the percentage of successful responses (Figure 5(a), pre: [*F*_2,14_ = 0.28, *P* ≤ 0.7580], Figure 6(a), pre: [*F*_2,13_ = 0.42, *P* ≤ 0.6631], Figure 7(a), pre: [*F*_2,14_ = 0.61, *P* ≤ 0.5526]) and the total number of responses (Figure 5(b), pre: [*F*_2,14_ = 0.14, *P* ≤ 0.8667], Figure 6(b), pre: [*F*_2,13_ = 1,30, *P* ≤ 0.3037], and Figure 7(b), pre: [*F*_2,14_ = 2.68, *P* ≤ 0.1033]) were similar in all experimental groups.

### 3.2. Postablation Phase: Effectiveness of the Lesion

The lesions were large and homogenous in size and location and similar to the ones in previous studies from our group [19, 31–33] (Figure 3(a)). In all rats, the primary and secondary motor cortex areas (M1 and M2) were damaged. In some cases, the lesion extended slightly into the cingulate cortex, area 1, (Cg1) (Figure 3(b); a schematic representation of a cortical lesion by aspiration is shown in [19]).

The effectiveness of the lesion was tested on day 7 after ablation. ANOVA showed that significant differences existed in the percentage of successful responses between CV, LV7, and LGH7 groups (Figure 5(a) post, *F*_2,14_ = 16.06, *P* ≤ 0.0002). The same thing happened when comparing CV, LV14, and LGH14 groups (Figure 6(a) post, *F*_2,13_ = 35.22, *P* ≤ 0.0001), and CV, LV35, and LGH35 groups (Figure 7(a) post, *F*_2,14_ = 24.08, *P* ≤ 0.0001). While the percentage of successful responses was preserved in the sham-operated control group (CV), these successful responses significantly decreased in all lesion animals (the Bonferroni post hoc test, *P* ≤ 0.0001), as Figures 5, 6, and 7 show (A, post). The low percentage of successful responses was similar in all lesion animals. Some of these lesion animals changed their preferred paw, while others continued to use the preferred paw, but in all the cases, the number of successful responses clearly decreased. With regard to the total number of responses, global ANOVA showed that no significant differences existed between the different experimental groups (Figures 5, 6, and 7(b), post).

### 3.3. Treatment with GH Plus Rehabilitative Therapies

#### 3.3.1. Treatment with GH 7 Days Postlesion Conjointly with Rehabilitative Therapy Produces Relevant Functional Improvement of the Motor Impairment Induced by the Cortical Ablation

The control group and animals treated with GH or vehicle at 7 days after cortical ablation underwent the first period of rehabilitative therapy, commencing on day 8 postlesion, in daily sessions of 3 min, for 9 consecutive days (Figure 1*S*). As described, it consisted in the forced use of the impaired paw (preferred paw) by means of a bracelet fitted on the nonpreferred paw. Repeated ANOVA measures (group and session) showed significant differences between groups (*F*_2,14_ = 6.69, *P* ≤ 0.009, *η*^2^ = 0.489) (Figure 5(a), short-term rehabilitation). In addition, given that in all groups, the number of successful responses was increasing along the rehabilitative sessions, a significant session effect was observed (*F*_8,112_ = 9.36, *P* ≤ 0.0001, *η*^2^ = 0.401). On the contrary, the group x session interaction was not significant (*F*_16,112_ = 1.21, *P* ≤ 0.2668, *η*^2^ = 0.148). Partial ANOVA tests showed significant differences among groups from the third to ninth rehabilitation sessions. As expected, the percentage of successful responses of the control group (CV) was similar during all sessions of this rehabilitation period, excepting the two first sessions in which they were lower (most likely due to the newness of the bracelet) and therefore not statistically different from animals with cortical ablation (Figure 5(a)). This lack of differences between groups in the two first sessions of rehabilitation agrees with the low number of total responses in these first two days observed in all groups (Figure 5(b)). However, interestingly, the Bonferroni post hoc test revealed that animals treated with GH 7 days postablation (LGH7) increased the percentage of successful responses along sessions, which became normal at the end of this rehabilitation period, similar to the percentage of successful responses in controls and remarkably higher than in lesion animals treated with vehicle (Figure 5(a)).

The total number of responses was similar in all the experimental groups. Therefore, no significant differences were observed among them (*F*_2,14_ = 2.23, *P* ≤ 0.1435) (Figure 5(b)). However, since all groups progressively increased the number of total responses along the rehabilitative sessions, a clear session effect (*F*_8,112_ = 14.62, *P* ≤ 0.0001) was shown. The group x session interaction was not significant (*F*_16,112_ = 1.35, *P* ≤ 0.1783).

In the second period of rehabilitative therapy (Figure 1*L*, days 35 to 43 postablation) with the forced use of the impaired paw, global ANOVA demonstrated that significant differences existed among groups (*F*_2,14_ = 5.55, *P* ≤ 0.0168, *η*^2^ = 0.442). However, neither session effect (*F*_8,112_ = 1.10, *P* = 0.366, *η*^2^ = 0.073) nor group x session interaction (*F*_16,112_ = 1.02, *P* = 0.440, *η*^2^ = 0.127) was observed. The percentage of successful responses of animals treated with GH (LGH7) was similar to that of the control group in all 9 sessions, while no improvement was observed in lesion animals treated with vehicle on post hoc analyses (Figure 5(a), long-term rehabilitation). Thus, the functional improvement of the motor deficit reached in LGH7 animals at the end of the first rehabilitative period maintained an asymptotic value in all 9 sessions. With regard to the total number of responses (Figure 5(b)), global ANOVA showed no significant differences among groups (*F*_2,14_ = 0.43, *P* ≤ 0.654). Neither session effect (*F*_8,112_ = 0.61, *P* ≤ 0.764) nor group x session interaction (*F*_16,112_ = 0.40, *P* ≤ 0.977) was observed.

#### 3.3.2. Treatment with GH 14 Days after Cortical Ablation Does Not Induce Any Improvement

In this case, GH or vehicle treatment was administered on days 14 to 18 postablation, that is, after the beginning of the first rehabilitative therapy (Figure 1*S*). The first period of rehabilitation consisted in daily sessions of 3 min for 13 consecutive days: 4 rehabilitative sessions before treatment with GH or vehicle, 5 sessions conjointly with GH or vehicle treatment, and 4 rehabilitative sessions after being treated with GH or vehicle (Figure 1*S*). Global ANOVA (group and session) showed significant differences between groups (*F*_2,13_ = 22.09, *P* ≤ 0.0001, *η*^2^ = 0.773). In addition, a significant session effect was observed (*F*_12,156_ = 6.14, *P* ≤ 0.0001, *η*^2^ = 0.321), but the group x session interaction was not significant (*F*_24,156_ = 1.11, *P* ≤ 0.3369, *η*^2^ = 0.146). Partial ANOVA tests showed significant differences among groups from the third to the thirteenth rehabilitative sessions (*P* ≤ 0.005). Again, with the same exception of sessions 1 and 2, both animals treated with GH or vehicle on day 14 postablation showed a significantly lower percentage of successful responses than control animals on post hoc analyses (Figure 6(a)). However, animals treated with GH showed a small increase in the percentage of successful responses from session 5 to session 12, coinciding with GH treatment, which tended to be higher than in lesion animals treated with vehicle, although the differences did not reach statistical significance (Figure 6(a)). The total number of responses was similar in all the experimental groups. Therefore, no significant differences were observed among them (*F*_2,13_ = 1.90, *P* ≤ 0.1882) (Figure 6(b)). However, since all groups progressively increased the number of total responses along the rehabilitative sessions, a clear session effect (*F*_12,156_ = 16.06, *P* ≤ 0.0001) existed.

In the second period of rehabilitative therapy (days 35 to 43 postablation) (Figure 6(a)), global ANOVA showed significant differences among groups (*F*_2,13_ = 25.46, *P* ≤ 0.0001, *η*^2^ = 0.797). However, neither session effect (*F*_8,104_ = 1.02, *P* = 0.424, *η*^2^ = 0.073) nor group x session interaction (*F*_16,104_ = 1.12, *P* = 0.346, *η*^2^ = 0.147) was observed. Animals treated with GH (LGH14) showed that the tendency observed during the days of GH administration disappeared. Therefore, their percentage of successful responses was similar to that of lesion animals treated with vehicle, and it was significantly lower than in control animals on post hoc analyses (Figure 6(a)). Hence, GH treatment starting on day 14 postlesion only slightly increased the percentage of successful responses during the days when the hormone was given, but this increase was no longer observed after interrupting GH treatment. With regard to the total number of responses (Figure 6(b)), no significant differences among groups (*F*_2,13_ = 2.94, *P* ≤ 0.0882), session effect (*F*_8,104_ = 1.27, *P* = 0.267), or group x session interaction (*F*_16,104_ = 0.83, *P* = 0.6427) were observed.

#### 3.3.3. Treatment with GH 35 Days Postlesion Improves the Motor Impairment Induced by the Cortical Ablation

In this case, treatment with GH commenced on day 35 after the cortical ablation, coinciding with the second period of rehabilitation (Figure 1*L*). That is, in the first period of rehabilitation, animals in the LGH35 group had not received the GH treatment yet. In this first period of rehabilitation, global ANOVA showed significant differences among groups (*F*_2,14_ = 16.87, *P* ≤ 0.0002, *η*^2^ = 0.707). A significant session effect was observed (*F*_8,112_ = 7.89, *P* ≤ 0.0001, *η*^2^ = 0.360), but the group x session interaction was not significant (*F*_16,112_ = 0.91, *P* ≤ 0.5535, *η*^2^ = 0.116). Partial ANOVA showed significant differences among groups (*P* ≤ 0.0008). Lesion animals (LGH35, LV35) presented a significantly lower percentage of successful responses than control animals (CV), once again with the exception of sessions 1 and 2 (Figure 7(a)). However, both lesion groups showed a slightly increasing trend in the percentage of successful responses along this first rehabilitative period (Figure 7(a)). No significant differences were observed in the total number of responses among the three groups (*F*_2,14_ = 3.15, *P* ≤ 0.741) (Figure 7(b)). However, a session effect (*F*_8,112_ = 16.90, *P* ≤ 0.0001) was shown. No significant group x session interaction was observed (*F*_16,112_ = 1.67, *P* ≤ 0.0612).

In the second period of rehabilitative therapy (days 35 to 43 postlesion), coinciding with GH or vehicle treatment, animals were again obliged to use the paw affected by the cortical ablation. Global ANOVA demonstrated that significant differences existed among groups (*F*_2,14_ = 7.82, *P* ≤ 0.0052, *η*^2^ = 0.528) and also a session effect (*F*_8,112_ = 2.44, *P* ≤ 0.018, *η*^2^ = 0.149); however, no group-session interaction (*F*_16,112_ = 1.21, *P* = 0.271, *η*^2^ = 0.147) was observed. The Bonferroni post hoc test revealed that animals treated with GH on day 35 after ablation (LGH35) showed a significant improvement of the motor impairment (Figure 7(a)). Therefore, excepting in session 1, during the rest of rehabilitative sessions, the percentage of successful responses was similar to that in control animals (Figure 7(a)) and notably higher than in lesion animals treated with vehicle in which no improvements were observed (Figure 7(a)). This significant improvement induced by administering GH and providing rehabilitation 35 days postablation was similar to that observed in animals treated with GH plus rehabilitation at 7 days after lesion. With respect to the total number of responses, global ANOVA showed no significant differences among groups (*F*_2,14_ = 0.41, *P* ≤ 0.667) (Figure 7(b)). Neither session effect (*F*_8,112_ = 0.59, *P* ≤ 0.784) nor interaction session group (*F*_16,112_ = 0.57, *P* ≤ 0.894) was observed.

### 3.4.4. Animals Treated with GH Reexpress Nestin and Actin in the Motor Cortex of the Undamaged Hemisphere

Immunoblotting studies were carried out in motor cortex samples obtained 51 days after cortical ablation. In general, the results show that nestin expression in the motor cortex of the undamaged hemisphere was higher in animals treated with GH at 7, 14, or 35 days after the lesion (LGH7, LGH14, and LGH35) than in vehicle-treated animals (LV7/35, LV14) or sham-operated controls (CV) (Figure 4(a), Nestin). No differences were observed in nestin expression between vehicle-treated animals (LV14 and LV7/LV35) and controls (CV). We can highlight the fact that the rat treated with GH on day 35 after the lesion showed very high nestin expression in the motor cortex of the undamaged hemisphere (Figure 4(a), LGH35). This animal showed the highest functional improvement after motor cortex ablation compared with the other rats treated with GH. With regard to actin, the expression of this protein was similar in the control group (CV) and in vehicle-treated animals (LV14 and LV7/LV35). However, animals treated with GH (LGH7, LGH14, and LGH35) showed higher amount of actin expression, compared with samples from control and vehicle-treated animals (CV, LV14, and LV7/LV35) (Figure 4(a), actin).  Figure 4(b) shows the densitometric quantification of nestin and actin expression from images shown in Figure 4(a), and these values have been plotted on a graphic that shows the association of nestin and actin expression. The coefficient of correlation between nestin and actin (*R* = 0.953) indicates that there is a very high relationship between the expression of these proteins. In fact, in some animals treated with GH, a strong correlation was observed between nestin and actin expression. Thus, samples from animals treated with GH show higher expression of nestin and actin (red underlined ellipse) than samples from control or vehicle-treated animals (green underlined ellipse). As it was mentioned above, the animal treated with GH on day 35 after the lesion showed the highest nestin and actin expression in the motor cortex of the undamaged hemisphere (Figure 4(b), LGH35), with good correlation with its functional improvement.

## 4. Discussion

We previously described in rats, for the first time, that after a severe lesion of the frontal motor cortex that produces a marked deficit in fine motor skills, early treatment with GH followed by rehabilitative therapy enabled the development of compensatory mechanisms in the contralateral undamaged hemisphere that allowed the functional recovery of the motor deficits resulting from the cortical ablation [19]. In that study, we also found that GH-treated animals showed a significant increase of nestin-positive cells in the intact contralateral motor cortex. This finding was also described for the first time, and we related it to the injury and the hormone since it was not found in vehicle-treated rats or sham-operated controls [19].

In this study, we tried to go further in the knowledge of these compensatory mechanisms and to know whether or not there is a window of time in which GH and rehabilitation are able to produce significant improvements after a severe cortical lesion. Our results demonstrate that GH plus rehabilitation treatment at day 7 or day 35 after the lesion lead to a remarkable functional improvement of the motor impairment induced by the lesion. By contrast, GH treatment on day 14 postablation did not induce any functional recovery.

Severe lesions of the motor cortex produce very important effects on the normal motor activity, affecting both the planning and organization of voluntary movements and their execution. The reason behind these effects is related to the limited capacity of the adult brain for self-repair after neuronal loss produced by an injury. In the case of cortical lesions, the alterations of axonal wirings produced by the trauma quickly led to a permanent functional impairment that caused severe behavioral deficits [31–33]. However, it is well known that any damage to the adult central nervous system generates adaptative brain plasticity. In fact, the adult brain is structurally dynamic [35–37], dendritic spines dynamically turn over in the adult brain [37, 38], and learning novel tasks are associated with a further increase in spine turnover [38]. Structural alterations that occur as a result of focal lesions in the brain have been identified, showing changes in axonal branching and growth [28]. Previous studies showed plasticity of the dendritic arborization in the cortex contralateral to a cortical lesion, and this is associated with improved skill in the limb unaffected by the lesion [39–41]. Another study has shown that rehabilitation can increase the numbers of dendritic spines and dendritic complexity in the cortical hemisphere contralateral to the brain lesion [42]. However, rehabilitation required several weeks of intensive training and practice of the impaired function, with rats undergoing 300–500 individual trials in order to regain about 60% of their prelesion skilled grasping ability [43]. This is concordant with our data in this study in which rats receiving only rehabilitation during a relatively short period of time were unable to reach any significant improvement in the paw affected by the cortical motor cortex ablation.

Animal studies indicate that many of the genes and proteins that are important for neuronal growth, synaptogenesis, and the proliferation of dendritic spines are expressed at their highest levels during early brain development and decline appreciably during ageing [44]. However, the second limited period of increased expression of these genes can be seen after a brain damage, such as what occurs in a stroke [45–47]. It seems that these new genetical reexpressions appear as an attempt to repair the damage occurred, since they have been seen surrounding the perilesional zone [48]. In spite of this, cases of significant damage, in which the repair is impossible because of the severity of the lesion, may lead to structural remodeling in some regions of the contralateral hemisphere [49, 50]. This agrees with our data in the present work and in our previous study [19]. We found remarkable nestin and actin reexpression in the contralateral undamaged frontal motor cortex in rats treated with GH and rehabilitation, but it was quite lower in animals receiving vehicle or in sham-operated controls.

Nestin is a class VI intermediate filament protein that, among other neural cells in the developing and adult central nervous system, was thought to be expressed exclusively in uncommitted neural progenitor cells (NPCs) and in endothelial cells [51–54], but it is also expressed in reactive astrocytes, especially after a brain injury [55]. After NPCs differentiate, nestin expression is usually replaced by the expression of neuronal or glial-specific markers. Since in our previous work, we detected remarkable nestin reexpression in the intact contralateral motor cortex of lesion animals treated with GH, but not in animals given vehicle or in sham-operated controls [19], and we assumed that this reexpression of nestin was related both to GH treatment and functional recovery. In that study, cells expressing nestin had the phenotype of neurons, but nestin immunoreactivity was detected in synaptic terminals outlining cell bodies of unlabeled neurons [19]. Therefore, it could be possible that these nestin-expressing neurons were already present and commenced to reexpress nestin after GH treatment and rehabilitative therapy, perhaps because of increased GH-receptor (GHR) expression and/or plasticity events leading to the remodeling of the undamaged contralateral motor cortex after the cortical ablation. We did not analyze whether GHR expression was upregulated in the contralateral intact cortex, but it has been recently described that during recovery from brain injury, there is an upregulation of GHR that may play a role in neuronal arborization and glial proliferation in the injured cortex [56].

The role of nestin in the central nervous system is not well known yet. It has been suggested that there is a selective distribution of nestin-expressing neurons in the cholinergic basal forebrain and regions of the brain involved in higher-order cognitive functions such as attention, learning, and memory, leading to the speculation that cell cycle and/or plasticity-related events may be involved in the expression of nestin by these neurons [26]. However, the authors themselves ruled out the hypothesis that in the normal brain the presence of nestin-expressing neurons may be due to the fact that these neurons had reentered the cell cycle and divided or that they arose from the division of another cell [26]. However, things may occur differently in an injured brain, as demonstrated by Nakatomi et al. [57]; their results, together with a number of other studies, showed that adult neural progenitors proliferate in situ in response to various insults, including trauma [58].

In mitotically active cells, nestin plays an important role, regulated by phosphorylation, in the assembly and disassembly of intermediate filaments, which contributes to remodeling the cytoskeleton of the cells. Nestin preferentially forms heterodimers with vimentin and alpha-internexin, possibly because these heterodimers are more stable than nestin homodimers. This is suggested by the fact that nestin contains a short N-terminal, a domain known to be essential for filament protein assembly [59] which inhibits filament formation *in vitro* when present at concentrations greater than 50% [60]. Thus, nestin may aid in linking intermediate filaments with each other, and with microtubules and microfilaments via the long C-terminal domain of nestin. Throughout the cell cycle, nestin colocalizes with the intracellular reorganization of vimentin filaments and aggregates and is essential for the mitotic disassembly of vimentin [61]. This suggests a role for nestin-mediated reorganization of the cytoskeleton during mitosis that is mediated in part by the upregulation of phosphorylation of Thr^316^ by cdc2 kinase [62].

Therefore, it seems logical to assume that the increased nestin reexpression we observed in the intact contralateral motor cortex of lesion animals treated with GH is related to a structural remodeling of the cytoskeleton triggered by severe cortical damage and the administration of GH together with rehabilitation in order to develop compensatory mechanisms leading to the functional improvement observed in two groups (LGH7 and LGH35) of these animals, but not in LGH14 group, in spite of the fact that increased nestin reexpression was also observed in them.

With regard to actin expression, it followed a pattern of expression parallel to that of nestin in lesion animals treated with GH and rehabilitation, remarkably higher than in lesion animals given vehicle or sham-operated rats. In fact, a strong correlation was observed between nestin and actin expression in some animals treated with GH.

Actin is a multifunctional protein that forms microfilaments and participates in many important cellular processes, including cell motility, cell division, vesicle and organelle movements, cell signaling, and maintenance of cell junctions and cell shape. Dendritic spines are actin-rich protrusions from the dendritic shaft, considered to be the locus where most synapses occur, as they receive the vast majority of excitatory connections in the central nervous system (CNS). Since changes in spine shape and size are correlated with the strength of excitatory synapses, spine morphology directly reflects spine function [63]. Actin has been reported to be present in cell nuclei (where it regulates the transcription of several genes) and cell membranes. Although actin is one of the most evolutionary conserved eukaryotic proteins, it is expressed in mammals as at least six major different isoforms characterized by electrophoresis and amino acid sequence analysis. Four of them represent the differentiation markers of muscle tissues, and two are found in virtually all nonmuscle cells.

There are three *α*-actins (*α*-skeletal, *α*-cardiac, and *α*-smooth muscle), one *β*-actin (*β*-nonmuscle), and two *γ*-actins (*γ*-smooth muscle and *γ*-nonmuscle). Since actin isoforms show >90% overall sequence homology, we decided to use an anti-actin polyclonal antibody addressed against the N-terminal domain of the molecule, instead of a more specific *β*-actin antibody. The *β*-actin gene has been considered a housekeeping gene in the CNS; however, its expression changes according to external or internal factors. By using the N-terminal anti-actin polyclonal antibody, we attempted to detect by immunoblotting the total expression of different actin isoforms in the brain homogenates: *α*-smooth muscle actin (brain vessels induced to proliferate by the injury, GH, and rehabilitation), *β*-actin (neural cells), and the two isoforms of *γ*-actins (brain vessels and neural cells).

As it happened with nestin, the amount of actin detected by immunoblotting in the homogenates of the intact contralateral motor cortex was notably higher in GH-treated lesion animals than in rats treated with vehicle or sham operated, suggesting that this increased expression of actin was related to the development of compensatory mechanisms in the undamaged cortex for functional recovery. Moreover, as described before, the animal that showed the highest expression of nestin and actin (LGH35) achieved the highest functional improvement after the severe motor injury was induced. Therefore, it is likely that the increased expression of nestin and actin in the contralateral hemisphere reflects compensatory mechanisms that appear after the injury and GH plus rehabilitation for the functional recovery of the affected paw.

However, interestingly, despite the fact that nestin and actin also increased in LGH14 animals, these were unable to reach a significant number of successful responses in the paw-reaching-for-food task. In this case, short-term rehabilitation commenced at the same day after the lesion than in the other groups, but GH treatment (on day 14 postablation) commenced at the end of the “critical period of time” in which maximal efficiency during rehabilitative therapies could be obtained (5 to 14 days after injury) [20] (Figure 1*S*). In this LGH14 group, just after initiating GH administration, a tendency to increase the number of successful responses was observed, but it did not reach statistical significance and it disappeared once GH administration was interrupted (Figure 6(a)).

It has been shown that after a stroke, in rats, soon a number of positive factors are induced in the peri-infarct region [48]; among them a number of growth factors have been described [45, 64, 65]. These factors might facilitate the sprouting of new axons [27, 66, 67] and support the increased elaboration of dendrites and spines [39, 68]. However, balancing these positive signals, there is an induced expression of negative factors that either inhibit outgrowth or repel sprouting axons. Among these inhibiting factors, the protein NOGO-A [69–71] and extracellular matrix factors such as chondroitin sulphate proteoglycan [72] play a pivotal role. Most growth-inhibitory genes tend to be upregulated gradually towards the end of the “critical period of rehabilitation” (see [48], for a more detailed explanation). Although these positive and negative effects on cortical regeneration only have been seen in the zones surrounding the lesion, it is likely that they may affect other areas of the brain (i.e., the contralateral undamaged frontal motor cortex). On these bases, the lack of significant improvements observed when GH was given 14 days after the cortical ablation could be explained by the presence of these negative factors that oppose to regeneration. The detection of clear increases in the expression of nestin and actin in these LGH14 animals is not against this assumption; while cells try to remodel their cytoskeleton to establish new circuits, they could not be set correctly or there are not enough of them. Further studies will reveal whether administering GH together with rehabilitation for a longer time after this critical period of time in which no improvements are observed will produce significant recoveries, similar to those observed in LGH7 and LGH35 animals.

In summary, it is interesting to highlight here that despite the long time elapsed since the lesion was produced, improvements in LGH35 animals were similar to those observed in LGH7 animals. This indicates that there is not a plateau in rehabilitation after a brain injury and agrees with recent data from our group [17], showing that despite the time elapsed after the brain injury and its severity, GH plus rehabilitation led to an almost complete recovery of the functionality of a young man affected by a plane crash [17]. Moreover, the fact that in this case the right hemisphere had been virtually lost in its entirety agrees with the idea that GH and rehabilitation lead to the development of compensatory mechanisms in the undamaged contralateral hemisphere, as we observed in this study. Therefore, if there is a time window during which GH plus rehabilitation cannot exert their positive effects on the brain repair after an injury, then this time window seems to be restricted in duration. Further studies will clarify this concept.

## 5. Conclusions

Our data allow us to conclude that GH is a very important factor in the repair of injured brains, but its administration has to be followed by immediate rehabilitation or accompanied by parallel rehabilitative therapy, in order to achieve functionally significant improvements. In the case of severe brain injuries, it is likely that GH administration and rehabilitation induce significant nestin and actin reexpression in the undamaged contralateral motor cortex. The amount of these reexpressed proteins seems to play a key role in the remodeling of the cytoskeleton of the cells and thus enable the development of compensatory brain plasticity which is responsible for the functional improvements observed. It remains uncertain whether GH administration together with rehabilitative therapy for a longer time after this critical period of time, in which no observed improvements would counteract the opposite negative effects of brain factors known to be released during this time. From our study, we cannot conclude whether there is a time window in which GH effects are counteracted by negative factors or it is a matter of doses. Lastly, our data indicate that there is not a plateau in recovery from a brain injury. Efforts have to be made to continue rehabilitative therapies after a brain injury beyond the time established in which no more recovery can be achieved.
